# Descriptive Evaluation and Accuracy of a Mobile App to Assess Fall Risk in Seniors: Retrospective Case-Control Study

**DOI:** 10.2196/16131

**Published:** 2020-02-14

**Authors:** Sophie Rabe, Arash Azhand, Wolfgang Pommer, Swantje Müller, Anika Steinert

**Affiliations:** 1 Lindera GmbH Berlin Germany; 2 Hochschulmedizin Freie Universität - Charité Berlin/Kuratorium für Dialyse und Nierentransplantation Neu-Isenburg Germany; 3 Charité – Universitätsmedizin Berlin, corporate member of Freie Universität Berlin, Humboldt-Universität zu Berlin, and Berlin Institute of Health; Geriatrics Research Group Berlin Germany

**Keywords:** falls, seniors, fall risk assessment, app, mHealth, retrospective cohort study, discriminative ability

## Abstract

**Background:**

Fall-risk assessment is complex. Based on current scientific evidence, a multifactorial approach, including the analysis of physical performance, gait parameters, and both extrinsic and intrinsic risk factors, is highly recommended. A smartphone-based app was designed to assess the individual risk of falling with a score that combines multiple fall-risk factors into one comprehensive metric using the previously listed determinants.

**Objective:**

This study provides a descriptive evaluation of the designed fall-risk score as well as an analysis of the app’s discriminative ability based on real-world data.

**Methods:**

Anonymous data from 242 seniors was analyzed retrospectively. Data was collected between June 2018 and May 2019 using the fall-risk assessment app. First, we provided a descriptive statistical analysis of the underlying dataset. Subsequently, multiple learning models (Logistic Regression, Gaussian Naive Bayes, Gradient Boosting, Support Vector Classification, and Random Forest Regression) were trained on the dataset to obtain optimal decision boundaries. The receiver operating curve with its corresponding area under the curve (AUC) and sensitivity were the primary performance metrics utilized to assess the fall-risk score's ability to discriminate fallers from nonfallers. For the sake of completeness, specificity, precision, and overall accuracy were also provided for each model.

**Results:**

Out of 242 participants with a mean age of 84.6 years old (SD 6.7), 139 (57.4%) reported no previous falls (nonfaller), while 103 (42.5%) reported a previous fall (faller). The average fall risk was 29.5 points (SD 12.4). The performance metrics for the Logistic Regression Model were AUC=0.9, sensitivity=100%, specificity=52%, and accuracy=73%. The performance metrics for the Gaussian Naive Bayes Model were AUC=0.9, sensitivity=100%, specificity=52%, and accuracy=73%. The performance metrics for the Gradient Boosting Model were AUC=0.85, sensitivity=88%, specificity=62%, and accuracy=73%. The performance metrics for the Support Vector Classification Model were AUC=0.84, sensitivity=88%, specificity=67%, and accuracy=76%. The performance metrics for the Random Forest Model were AUC=0.84, sensitivity=88%, specificity=57%, and accuracy=70%.

**Conclusions:**

Descriptive statistics for the dataset were provided as comparison and reference values. The fall-risk score exhibited a high discriminative ability to distinguish fallers from nonfallers, irrespective of the learning model evaluated. The models had an average AUC of 0.86, an average sensitivity of 93%, and an average specificity of 58%. Average overall accuracy was 73%. Thus, the fall-risk app has the potential to support caretakers in easily conducting a valid fall-risk assessment. The fall-risk score’s prospective accuracy will be further validated in a prospective trial.

## Introduction

Falls have a high prevalence among seniors, with 1/4 seniors aged 65 and above experiencing one fall per year [1-3]. Fall rates in nursing homes are higher than fall rates in the community. Rubenstein et al [4] provided an incidence rate of 1.7 falls per person, per year, for nursing facilities compared to an incidence rate of 0.65 falls per person, per year, for older people living in the community. The prevalence of fall-related injuries has also been found to increase with age [5]. Around 10-15% of falls result in a fracture [6]. Furthermore, fall-associated fractures among older people are significantly related to morbidity and mortality.

Due to demographic changes associated with an aging population, the number of falls among older adults is expected to rise considerably. A recent study even reported an increased rate of death from falls. These researchers investigated data from people who died as a result of a fall. The data showed that the rate of deaths from falls increased by an average of 3.0% per year during 2007-2016 [7]. Therefore, effective fall prevention strategies should be promoted and implemented.

Fall-risk assessment is a complicated task. Current scientific evidence suggests that a multifactorial fall-risk assessment, including an analysis of mobility as well as extrinsic and intrinsic risk factors, is crucial [1-3,8,9]. In Germany, the assessment of fall risk according to guidelines defining risk assessment and fall prevention procedures is mandatory in inpatient care [10]. However, this process includes a time-consuming and challenging subjective analysis of the patient’s mobility status and a multitude of additional individual risk factors.

Thus, a smartphone-based application, Lindera Mobilitätsanalyse (Lindera GmbH, Berlin, Germany), was developed to facilitate fall-risk assessment. As a stand-alone software, this app enables nursing staff to perform a structured fall-risk assessment that conforms to regulatory standards [10].

Further app-based, fall-risk assessment tools have been identified in the literature [11-14]. One such fall-risk assessment app is the Aachen Fall Prevention Scale. This app is a self-assessment tool that consists of a simple questionnaire with a balance test that is self-assessed and evaluated. The app seeks to raise older adults' awareness of their fall risk. The Aachen Fall Prevention App was found to have a pooled sensitivity of 57.0% and a specificity of 76.7% [14]. A further fall-risk app is called Steady. This app consists of a health history questionnaire and five progressively more challenging mobility tasks to measure individual fall risk. This app was found to be highly usable among older adults but has not yet been evaluated in terms of validity, although the authors mention testing the app’s validity as the next step for future research [11]. Both apps focus on individual seniors as users and assess mobility with challenging postural stability tasks. The Lindera mobility analysis was designed to support nursing staff and is the first fall-risk app that enables nurses to perform an objective, structured, fall-risk assessment that conforms to regulatory standards.

Fall-risk assessment tools should accurately discriminate fallers from nonfallers. Diagnostic accuracy relates to the fall-risk score’s ability to discriminate between faller and nonfaller status. The discriminative performance of fall-risk assessments has frequently been quantified using measures such as sensitivity, specificity, and the area under the curve (AUC). The validity of each assessment tool should be evaluated to interpret the results correctly. Currently, the diagnostic test accuracy of most existing fall-risk assessment tools appears to be modest [1,15]. Overall diagnostic accuracy results must incorporate relative misclassification costs to account for the fact that false-negative and false-positive results are rarely clinically equivalent [16]. As there is always a trade-off between sensitivity and specificity, it is essential also to include the receiver operating curve. Measures of test accuracy can be limited by their dependence on the prevalence of an outcome. Measures that perform well among people for whom there is a strong suspicion that they have the condition being assessed (ie, the prevalence is close to 50%) will nearly always perform poorly in trying to identify people when the prevalence is low [17].

This paper aimed to study the discriminative ability of the fall-risk score with the aid of learning models. These models were evaluated based on relevant performance metrics, such as the receiver operating curve and its area under the curve, using a real-world dataset containing subjects with and without a previous fall history.

## Methods

### Study Design and Study Participants

The study was designed as a retrospective analysis of the Lindera user database. All study participants agreed to the collection of data presented in this publication by signing the terms and conditions for the use of Lindera as well as a written informed consent form. Lindera is compliant with the European Union General Data Protection Regulation. All data analyzed for the study were anonymized for statistical analysis.

The study sample consisted of seniors who completed a fall-risk assessment via the app between June 2018 and May 2019 and uploaded their data to the company’s user database. The app only provides analyses for customers who have signed a data processing contract. The company’s customers include nursing homes, outpatient nursing services, care support centers, and daycare institutions. Seniors were recruited and informed by nursing staff in these institutions.

To assure data quality and homogeneity among the study population, only participants aged 65 and above where analyzed, as this is seen as a relevant cut-off age for a higher occurrence of falls [18]. Furthermore, only seniors who provided information about their fall status over the last 12 months (faller or nonfaller) were included. Fall status was either self-reported or reported by nursing staff completing the assessment.

Due to the nonexperimental, retrospective, and anonymized study design, no ethical approval was needed.

### Description of the Fall-Risk Score and Use of the App

Nurses can analyze a senior’s mobility according to the Tinetti test criteria [19] via a smartphone camera and an underlying computer vision algorithm . This underlying algorithm is based on a combination of the convolutional pose machine (two-dimensional joint detection) and the VNect algorithm (three-dimensional joint and skeleton detection) [20,21]. Two procedures must be completed to provide a fall-risk assessment, the first of which is a smartphone-based video analysis, where a member of the nursing staff captures the senior’s gait. The senior has to sit on a chair, stand up and walk about 3 meters toward the camera, then turn and walk back again. Seniors had to be able to perform this mobility test as a prerequisite for completing the full assessment. The use of walking aids was allowed (eg, walker, cane). After the mobility test, a questionnaire assessing further fall-risk factors had to be completed within the app. The questionnaires were either self-assessments or completed with the help of nursing staff. Only fully completed and uploaded assessments were analyzed. Nursing staff received a standardized training course by the Lindera customer success team on how to use the app and the questionnaire.

Every risk factor within the analysis is considered in the fall-risk score, which is a metric scale ranging from 0-100 points. Per validated fall-risk models that have shown a good diagnostic test accuracy [1,22] (STRATIFY [St. Thomas's Risk Assessment Tool In Falling Elderly Inpatients] Fall Risk Assessment Tool, Hendrich Fall Risk Model II, Downton Fall Risk Assessment), nine of the risk factors are given a double weighting (limited mobility, dizziness, visual and acoustic impairment, medication, cognitive impairment, depression, urge incontinence, fall history, and restlessness). Further evidence-based risk factors are weighted once (mobility-limiting comorbidities, foot disorders, comorbidities that lead to syncope, fear of falling, use of walking aids, and environmental hazards). Fall events were identified with an app-based question asking whether the senior had experienced a fall during the last 12 months. For a detailed description, please refer to the documentation of the scientific approach underlying the app [23]. To offer prevention strategies, an individualized fall prevention plan was provided with every analysis. The preventative measures were derived from an evidence-based recommendation database [23]. An individual fall-risk assessment and prevention plan were sent to each customer within 24 hours after they uploaded the analysis. An example prevention plan can be found in Multimedia Appendix 1.

The fall-risk score assessment was completed using an app named Lindera Mobilitätsanalyse. The nursing staff was able to download the app for iOS (App Store) or Android (Google Play Store) mobile devices. The app was free to download, but to get the analysis results, care providers and study participants had to sign a data processing contract with the company and a declaration of consent following data protection law. The collaborating care provider covered the analysis costs. In Germany, care institutions have a prevention budget that provides a legal basis for them to fund appropriate solutions.

### Data Collection

All data analyzed in this study were entered by the app’s users and stored on company servers hosted by Deutsche Telekom and located in Bonn, Germany. The Chief Technology Officer of Lindera and backend employees had access to the database and extracted anonymized data for scientific evaluation. No identifiable patient information has been or will be shared.

### Statistical Analysis

#### Descriptive Statistics

All statistical analyses were conducted using Python version 3.6.8 (Python Software Foundation, Wilmington, United States) with the aid of the Pandas library version 0.24.2. All modeling research was done using the scikit-learn machine learning library for Python, version 0.20.3. Python is widely used for conducting statistical analyses [24,25]. Descriptive statistics, including means, standard deviations, and distributions, were provided for all study variables and compared across groups (fallers vs nonfallers). To test for significant differences (*P*<.05) between groups, a two-sample, two-tailed *t* test was applied for metric variables, and a chi-squared test was applied for categorical data.

#### Model-Based Statistics

The ability to discriminate between fallers and nonfallers using the fall score feature alone was analyzed, prioritizing a high sensitivity. One of the best performance metrics for quantifying the accuracy of medical diagnostic tests, like the one considered here, is the receiver operating characteristic (ROC) [26-28].

To determine the ROC for the two-class classification model, we first calculated the confusion matrix for a predefined test dataset. Secondary performance metrics, like sensitivity, specificity, accuracy, and precision, can be easily calculated from the confusion matrix. Detailed descriptions of the concepts of the ROC, the confusion matrix, and secondary performance metrics, with a clear focus on the sensitivity-specificity trade-off, can be found in the supplementary materials section (see Multimedia Appendix 2).

In this study, we investigated and compared the following five models: Logistic Regression, Gaussian Naive Bayes, Gradient Boosting, Support Vector Classification, and Random Forest Classification. The primary reason for choosing these models was that they exhibit good selection capabilities over multiple model types and are well studied in applications of machine learning in the medical field [29,30] (for more details about the general theory and application of machine learning algorithms, see books by Hastie et al, Dangeti, and Bowles [31-33].) In all models used in the analysis, the fall score was the only independent variable used to predict the target output for each subject in the dataset, namely their classification into the nonfaller (0) or the faller (1) group.

The modeling pipeline was as follows. First, we partitioned the dataset into two subsets via a stratified random split. A total of 85% of the dataset went into a training-validation set (205 subjects) and 15% into a test set (37 subjects). We chose to perform a stratified split in order to ensure that the two classes had the same distribution in both subsets. Next, we performed a stratified k-fold cross-validation (with k=8 splits) [34-36] on the training-validation subset, with the test set remaining untouched to enable the later evaluation of the final models on a real-world dataset. Details of the k-fold cross-validation can be found in the supplementary materials section (see Multimedia Appendix 2). The k-fold cross-validation helped us to identify a final form for each model and its mean cut-off probability for optimizing the sensitivity-specificity trade-off. We then trained these final models on the complete training-validation subset and calculated performance metrics based on the test dataset.

## Results

### Descriptive Statistics

The sample had a mean age of 84.6 years (SD 6.7), and 169/242 participants (69.9%) were female. A total of 139 seniors (57.4%) reported no previous falls (nonfaller), whereas 103 seniors (42.5%) reported at least one fall event in the last 12 months. There was no statistical difference in age (*P*=.87) or gender (*P*=.41) between fallers and nonfallers. Overall, 131 seniors (54.1%) were living in nursing homes, 34 (14.1%) in assisted living facilities, and 77 (31.8%) at home. There were 40 seniors (16.5%) who lived at home and received outpatient care.

**Figure 1:**
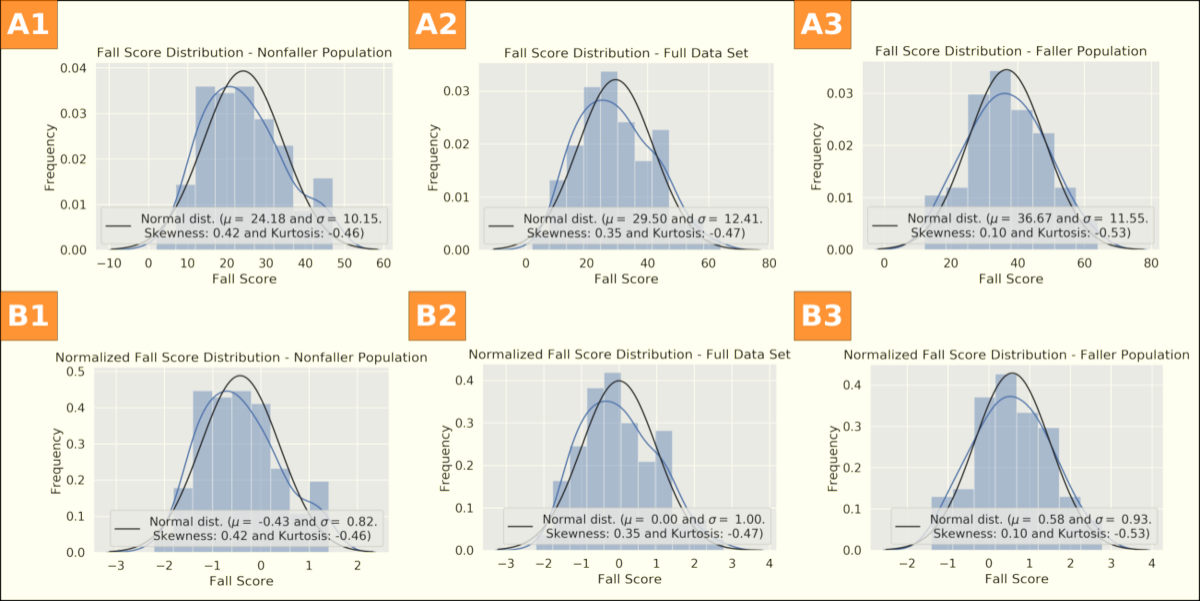
Fall-risk score histograms. Row A shows histograms for nonnormalized fall scores and Row B for standard-scaled fall scores. A1 and B1 show the nonfaller subgroup, A2 and B2 show the full dataset, and A3 and B3 show the faller subgroup.

The average fall-risk score was 29.5 points (SD 12.4). Fallers had an average fall-risk score of 36.7 (SD 11.6), while nonfallers had an average fall-risk score of 24.0 (SD 10.2). All analyzed subgroups showed a normal distribution (see Figure 1). There was a highly statistically significant difference in fall-risk scores between fallers and nonfallers (*P*<.001).

We show the standard-scaled fall score distributions in Figure 1. The normalized fall score distribution resembles a Gaussian distribution with a zero mean and standard deviation of one. Accordingly, the mean for the nonfaller subgroup is negative, while the mean for the faller subgroup is positive.

Skewness and kurtosis factors of the distributions are also shown in Figure 1. Both are in a range corresponding with a normal distribution (skewness between –0.5 and +0.5).

### Model-Based Statistics

The results of the k-fold stratified cross-validation are shown in Table 1. The average sensitivity was around 85.0%. The average optimal cut-off probability was 0.32 (SD 0.06), and the corresponding cut-off fall score was 27.3 points (SD 3.4). A subject at or above that fall score value was classified into the faller subgroup on average.

**Table 1:** Results of the k-fold stratified cross-validation study (k=8).

	LR^a^ model	GNB^b^ model	GB^c^ model	RF^d^ model	SVC^e^ model	Overall average (SD)
AUC^f^ (SD)	0.76 (0.09)	0.76 (0.09)	0.75 (0.09)	0.74 (0.07)	0.74 (0.09)	0.75 (0.08)
Sensitivity, % (SD)	85.0 (4.0)	85.0 (4.0)	85.0 (5.0)	85.0 (5.0)	84.0 (4.0)	85.0 (4.0)
Specificity, % (SD)	49.0 (10.0)	49.0 (10.0)	54.0 (17.0)	50.0 (8.0)	51.0 (15.0)	50.0 (12.0)
Accuracy, % (SD)	71.0 (6.0)	71.0 (6.0)	68.0 (7.0)	66.0 (8.0)	71.0 (6.0)	70.0 (7.0)
Precision, % (SD)	56.0 (5.0)	56.0 (5.0)	59.0 (8.0)	56.0 (4.0)	56.0 (6.0)	57.0 (6.0)
Cut-off probability (SD)	0.31 (7.0)	0.29 (0.07)	0.38 (0.05)	0.34 (0.06)	0.27 (0.05)	0.32 (0.06)
Cut-off fall score points, mean (SD)	25.3 (3.3)	25.0 (3.0)	29.5 (3.4)	29.4 (6.0)	27.4 (1.4)	27.3 (3.4)

^a^LR: Logistic Regression.

^b^GNB: Gaussian Naive Bayes.

^c^GB: Gradient Boosting.

^d^RF: Random Forest.

^e^SVC: Support Vector Classification.

^f^AUC: area under the curve.

In a final step, we considered the models with the best average cut-off probabilities as the optimal models. These optimal models were then trained on the full training-validation set (85% of the complete dataset), while test metrics were calculated on the remaining hold-out test set (15% of the complete dataset). Validation metrics for the individual models, together with the averages across all models, are presented in Table 2.

**Table 2:** Results of the test set metrics for the final models.

	LR^a^ model	GNB^b^ model	GB^c^ model	RF^d^ model	SVC^e^ model	Overall average (SD)
AUC^f^	0.9	0.9	0.85	0.84	0.84	0.86 (0.03)
Sensitivity, %	100.0	100.0	88.0	88.0	88.0	93.0 (6.0)
Specificity, %	52.0	52	62.0	56.0	57.0	58.0 (5.0)
Accuracy, %	73.0	73.0	73.0	70.0	76.0	73.0 (2.0)
Precision, %	62.0	62.0	64.0	61.0	67.0	63.0 (2.0)

^a^LR: Logistic Regression.

^b^GNB: Gaussian Naive Bayes.

^c^GB: Gradient Boosting.

^d^RF: Random Forest.

^e^SVC: Support Vector Classification.

^f^AUC: area under the curve.

**Figure 2:**
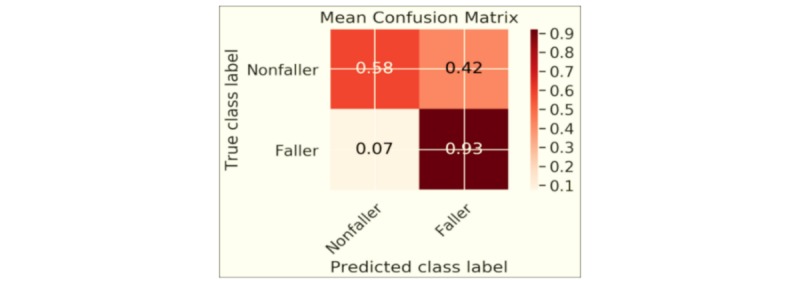
Confusion matrix averaged over all five models.

**Figure 3:**
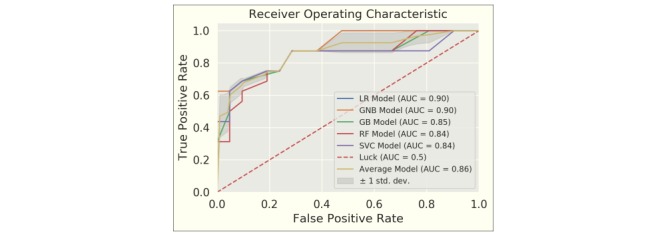
ROC curves and corresponding AUCs for the five models and the average over all five models.

Figures 2 and 3 illustrate the main results of the finalized models, evaluated on the hold-out test set. The average confusion matrix is shown in Figure 2 . The models are quite sensitive (93% of the faller subgroup correctly classified to the faller group). Figure 3 displays ROC curves for all five models together with the average ROC curve. The mean AUC over all models is 0.86, and we can observe that most model ROC curves are located one SD above and below the average ROC curve (the grey area in the ROC plot). The high average AUC indicates that the fall score had very good separability.

## Discussion

### Study Findings

The study’s main finding was that the fall-risk score exhibited a high discriminative ability to distinguish fallers from nonfallers across all six models evaluated. The models had an average AUC of 0.86, an average sensitivity of 93%, an average specificity of 58%, and an average accuracy of 73%. As discussed in the methods section, AUCs near 1 (0.8-0.9) indicate very good separability of the models and their corresponding features [26,37]. Thus, an average AUC of 0.86 indicates a very good discriminative ability of the fall score feature, which is further reinforced by the high average sensitivity of 93%.

Our results provide a descriptive evaluation of the designed fall-risk score for a sample of very elderly seniors with a mean age of 84.6 years old (SD 6.7). This high average age may be because more than half of the sample (54.3%) were nursing home residents. A total of 14.1% were living in assisted living facilities, and 16.1% received ambulant care. Thus, a large share of the investigated population was in high need of care. The high percentage of fallers (42.5%) in the sample may also be attributable to these demographic characteristics. There is currently only limited data on fall rates among seniors of very high age. Rapp et al [38] found retrospective one-year fall rates of 44.1% for women and 46.9% for men. Von Heideken Wågert et al [39] reported a retrospective one-year fall prevalence of 45% in a cohort of seniors above age 85. Similarly, van Bemmel et al [40] reported a fall rate of 44% for 85-year-old seniors. Moreover, 69.8% of the participants in the present study were female. This reflects the higher percentage of females in the elderly population, particularly at very high ages [41]. Hence, the sample seems to be representative of seniors of very great age for the discussed patient characteristics.

The average fall-risk score in this sample was 29.5 points (SD 12.4). The descriptive data analysis clearly shows that fallers had significantly higher fall-risk scores than nonfallers (*P*<.001). Moreover, the fall-risk scores in the evaluated groups were normally distributed, facilitating a good discriminative ability. This data can be used as reference values to judge and compare seniors’ fall-risk scores. Furthermore, the dataset continues to grow as the use of the fall-risk assessment app continues, and reference values with an even higher sample size will exist in the future. Reference values for different subgroups will also be made available as the sample size increases.

### Comparison With Prior Work

A large number of studies have evaluated the accuracy of fall-risk assessments [1,3,14,22,42]. Regarding AUC values, Lee et al [42] conducted a review of 31 studies and reported accuracy values for fall-risk assessments ranging from 0.62-0.89. More recently, Park et al [1] conducted a meta-analysis of 33 fall-risk assessment tools. They reported AUC values ranging from 0.76-0.97, sensitivity values ranging from 53%-89%, and specificity values ranging from 26%-90%. Based on criteria recommended by Olivier et al [43], fall-risk assessments with a sensitivity of ≥70% are considered acceptable. Park et al reported specificities under 60% for nearly all evaluated assessment tools. Furthermore, Rasche et al [14] conducted a meta-analysis reviewing the latest fall-risk assessment measures and reported a mean sensitivity range of 57.0%-90.0% and a mean specificity range of 30.6%-84.3%. Average AUC values for the included fall-risk assessments ranged from 0.69-0.90. Consequently, the newly developed fall-risk score presented in this study achieves accuracy measures that are comparable to established fall-risk assessments.

It must be stated that all of our evaluated models achieved a specificity below 70%. This means that there is a tendency to report a higher risk of falling. This, in turn, could affect the fall prevention strategies recommended by the app. However, a lower specificity can be tolerated due to the noninvasive nature of fall prevention strategies, which often address general health issues. In other words, given the noninvasive nature of fall prevention interventions, falsely diagnosing someone as high risk is considered less detrimental than falsely categorizing someone as low risk (which would result in falls not being prevented). The primary goal of a fall-risk assessment tool is to identify people at a high risk of falling to minimize the occurrence of falls. Accordingly, we conclude that if a fall-risk assessment tool has a high sensitivity, it achieves its primary goal, even though the specificity is low. Thus, although the specificity is not ideal, the overall performance of the fall-risk score and its sensitivity-specificity trade-off meet the specific requirements of a tool for fall prevention.

The available research on the accuracy of fall-risk assessment tools exhibits high interstudy heterogeneity [1,22,42]. Because falls are multifactorial, it should be noted that all fall-risk assessments have imperfect accuracy. It is highly improbable that a single fall-risk assessment tool will be able to accurately assess all individually relevant risk factors and risk factor combinations. Nonetheless, these risk assessment tools can offer valuable help to clinicians and nursing staff and facilitate the identification of at-risk seniors and suitable interventions. Oliver et al stated that identifying and modifying risk factors seems to be the optimal strategy to prevent falls, as opposed to focusing only on risk prediction, which may be inaccurate and will not in and of itself prevent patients from falling [44]. Therefore, the evaluated fall-risk score is provided in combination with a tailored prevention plan for every senior assessed. Furthermore, a metric fall-risk score enables the quantification of fall risk, which could help to evaluate the effects of prevention strategies.

To assist health care professionals in understanding the fall-risk score, we suggest a cut-off value. In a precision-sensitivity study, a cut-off value of 27.5 points (SD 4.5) was shown to offer the best combination of sensitivity and specificity. Thus, seniors with a score higher than 27.5 points (SD 4.5) can be classified as having a high fall risk and should be prioritized in the implementation of prevention strategies. However, this cut-off value should be seen as merely a preliminary recommendation. Evaluations of larger sample sizes with prospective data may lead to further adjustments in the recommended cut-off score.

### Limitations

This study’s limitations arise from its retrospective case-control study design, which makes it potentially vulnerable to selection bias. The potential for recall bias should also be considered. Recall bias refers to the increased likelihood that fallers will recall and report the presence of risk factors, whereas nonfallers are less likely to report risk factors [45]. Furthermore, this study evaluated data on retrospective fall status, which may have led to higher fall-risk scores among fallers. In other words, a past fall event may have led to higher values of the investigated risk factors (eg, limited mobility, fear of falling). These methodological issues will be addressed in further data analyses with a dataset that includes prospective data on fall status. A further methodological improvement could be the addition of a third group of frequent fallers. Frequent falls are associated with the most considerable risk of future falls [46] and could, therefore, provide insights about a high-risk population in need of the greatest support in terms of prevention strategies.

Moreover, there is a discussion in the fall-risk literature about the self-reporting of falls. One-year retrospective self-reporting of falls has been found to result in a slight underreporting [47,48]. Additionally, there is a need for a clear and simple definition of fall events from a methodological perspective [49]. The lack of a clear definition may have biased the assessment of fall events. A clear definition will become even more critical when the app is used without support from the nursing staff. Furthermore, our sample might not be representative of the broader population of older adults, and especially of community-dwelling older adults. Future research is needed to investigate the accuracy of the fall-risk score in further population segments.

### Outlook

The digital assessment of fall risk has the potential to objectify and improve fall-risk assessment and reduce the subjectivity introduced by human judgment due to biases, prior knowledge, experience, preferences, and limited capacities to absorb information.

Various researchers have concluded that the validity of current fall-risk assessment tools is not enough [1,3,15]. Therefore, new approaches are needed. As the fall-risk assessment app’s number of users grows, there is the potential to gain more in-depth insights from real-world data on the development of fall risk, fall-risk factors, different subgroups, and the effectiveness of fall prevention strategies based on large sample sizes. Gaining knowledge about effective fall-risk assessment and prevention in the geriatric population is critical considering current demographic challenges related to an aging population [50].

### Conclusion

The descriptive statistics provided can be used as comparison and reference values for users of the fall-risk assessment app. The fall-risk score showed a high discriminative ability to distinguish fallers from nonfallers in all the evaluated models. On average, the models exhibited good accuracy, excellent sensitivity, moderate specificity, and good AUC values. The fall-risk assessment app has the potential to support nursing staff in performing valid, systematic, and objective fall-risk assessments that can be used to identify relevant risk factors and implement multifactorial prevention strategies. The fall-risk score’s predictive validity will be further validated in future prospective trials, including larger sample sizes based on a growing real-world database.

## Appendix

Multimedia Appendix 1 Example prevention plan.

Multimedia Appendix 2 Details of model-based statistics.

## Abbreviations

AUC: area under the curve
ROC: receiver operating characteristic
STRATIFY: St. Thomas's Risk Assessment Tool In Falling Elderly Inpatients
