# Supplementary material for: Descriptive Evaluation and Accuracy of a Mobile App to Assess Fall Risk in Seniors: Retrospective Case-Control Study
Source: JMIR Aging. 2020 Feb 14;3(1):e16131. doi: 10.2196/16131 (PMC7055764; doi:10.2196/16131)
Supplement: Multimedia Appendix 1 [file aging_v3i1e16131_app1.pdf]

LINDERA

Mobility Analysis

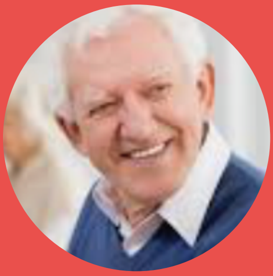

John Doe

John Does propensity of falling
Status: **February 2019**

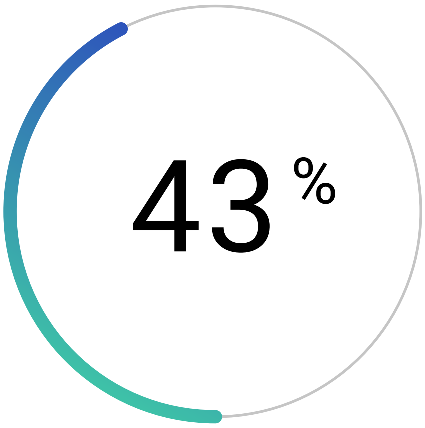

propensity of falling

Personality and character

Routines are very important to Mr. Doe. He values consistency and traditions. Occasionally, he tends to be nervous and irritable, but mostly he is satisfied and relaxed.

Mr. Doe appreciates being with other people. When dealing with other people, he pays more attention to his own needs.
Mr. Doe describes himself as careful and likes to plan ahead.
Responsibility and reliability are important to him.

- ✓ **traditionally**
- ✓ **dogmatic**
- ✓ **cautious**

Factor: **Functional impairments**

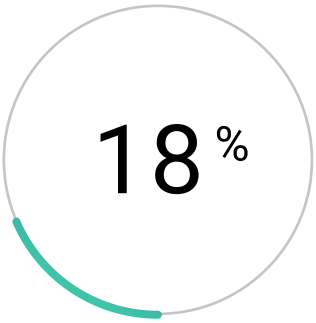

Sudden syncopes can have a negative impact on the risk of falling. Moreover, the patient has osteoarthritis, which can lead to gait and balance disorders.

In addition, back pain would also increase the risk of falling.

A physiological step height is ideally 2-4 cm, whereas the patient seems to have an even smaller stride length.

Physical pain has reduced the gait velocity, while the upper body is bent forward. In turn, this displaces the center of gravity and is associated with a higher fall risk.
Furthermore, the trunk is shifted strongly to the left and the time taken to get up is considerably slow.
Finally, the patient mostly looks down while walking, so this restricts the field of vision and the recognition of potential obstacles.

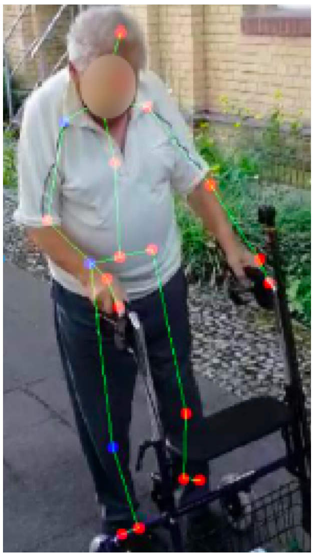

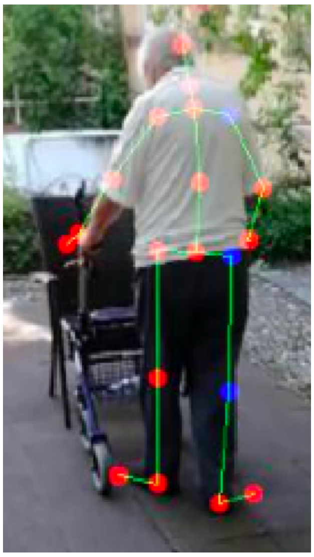

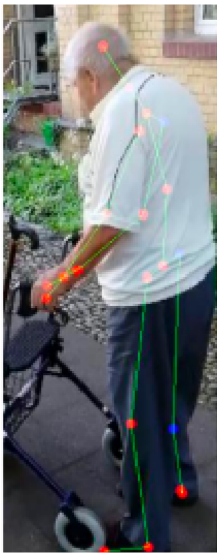

Factor: **Functional impairments**

Step length **Ø 13,26**

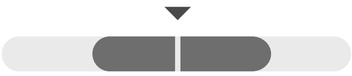

Links:
13,19 cm

Rechts:
13,34 cm

step height Ø **Ø 1,32**

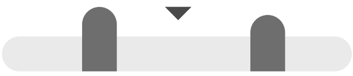

Links:
1,33 cm

Rechts:
1,31 cm

gait velocity

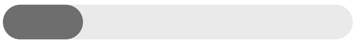

0,63 m/s

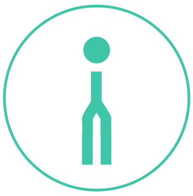

gait pattern:
symmetric

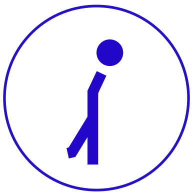

center of gravity:
shifted forward

15,3 °

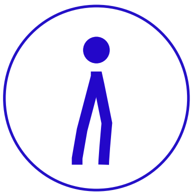

center of gravity:
shifted to the side

7,54 °

Recommendations:

- Promote physically active phases in your everyday life (for example, through walks and participation in exercise programs).
- Develop attention for your own gait pattern (lift your feet when walking clearly off the ground) and try to look straight
- Regular medical diagnosis and therapy planning for dizziness, osteoarthritis and back pain.

Factor: **sensory impairment and cognition**

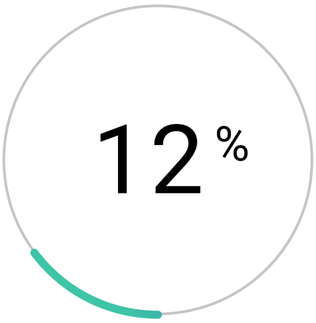

Severe visual impairments reduce the orientation in space and reduce perception. In addition, pronounced difficulties with hearing impede spatial orientation and balance.

Furthermore, excessive consumption of alcoholic beverages may increase the risk of falling and negatively affects existing diseases.

Recommendations:

- medical check up of impaired vision and hearing by a ophthalmologist/ optician
- medical check up of impaired hearing by a acoustician and if necessary regular use of the hearing aids
- Pay attention to a moderate alcohol consumption. For men 20 g / day are recommended (about two bottles of beer (0.3 l each) or two Glasses of wine (each 0.1 l) or two glasses of schnapps (0.03 l)).

Factor: **Diseases that lead to fainting and medications**

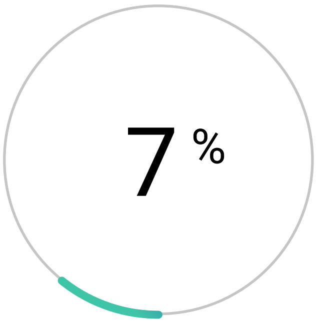

A sudden drop in blood pressure can be associated with a temporary loss of consciousness and potential injurious falls. The patient takes more than five different medications daily.

The high drug intake can lead to unforeseen adverse reactions and side effects.
In particular, cardiovascular medications can elevate the risk of falling.

Recommendations:

- Regular medical diagnosis and therapy planning for controlling and adjusting blood pressure.
- Perform simple exercises before getting up (for example, stretch and tighten tiptoes 10x or stretch arms over your head while clenching and opening your fists).
- regularly checkup of medication by your family doctor, especially if your medication is prescribed from various facilities
- Talk to your physician about the PRISCUS list. Discuss symptoms such as dizziness, double vision, tiredness or nausea with a nurse and with your doctor.

Factor: **walking aids and environmental hazards**

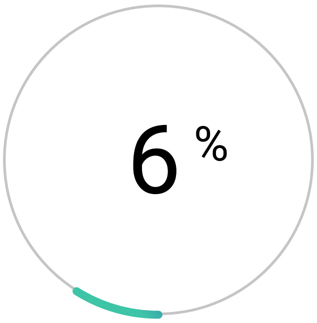

As walking aid, a walker is mentioned.
There are a number of risks associated with household items.

Often, households contain carpets and walking without firmly gripped slippers can significantly increase the risk of falling.

Recommendations:

- Adjust the settings of the walker: adjust the height of the level to match the level of your wrists. Make sure arms are only slightly flexed.
- If you feel uncertain about walking by yourself, use your walker consistently .
- Stick loose carpets, place stoppers under the carpets or completely remove the carpets.
- Wear closed and well-fitting shoes both inside and out (recommendation: shoes with flat, wider, hard and non-slip sole, possibly bandage shoes).

This analysis does not replace a medical diagnosis or therapy. The Lindera mobility analysis builds on the evidence-based and widespread Five-factor model of personality. This model has been reviewed and validated in numerous studies with more than 100,000 people and is based on an internationally recognized questionnaire. Further information regarding the background and scientific approach can be found here: https://www.lindera.de/wissenschaftlicheransatz/.
Please discuss the factors indicated by us with your doctor or nurse.

LINDERA
