# Supplementary material for: Descriptive Evaluation and Accuracy of a Mobile App to Assess Fall Risk in Seniors: Retrospective Case-Control Study
Source: JMIR Aging. 2020 Feb 14;3(1):e16131. doi: 10.2196/16131 (PMC7055764; doi:10.2196/16131)
Supplement: Multimedia Appendix 2 [file aging_v3i1e16131_app2.docx]

***B. Details of Model Based Statistics***

***B1. Receiver Operating Characteristics***

In our model-based study we considered the Receiver Operating Characteristic (ROC) [1–3] as best performance metric for quantifying the accuracy of medical diagnostic tests. Both the concept of a confusion matrix as well as the ROC are schematically represented in Fig. B1. Here, panel A displays the confusion matrix and its numeric elements. These elements in the matrix form a set of base metrics:

- TP (True Positive): Fallers, correctly classified as fallers.
- TN (True Negative): Non-fallers, correctly classified as non-fallers.
- FP (False Positive): Non-fallers, falsely classified as fallers.
- FN (False Negative): Fallers, falsely classified as non-fallers.


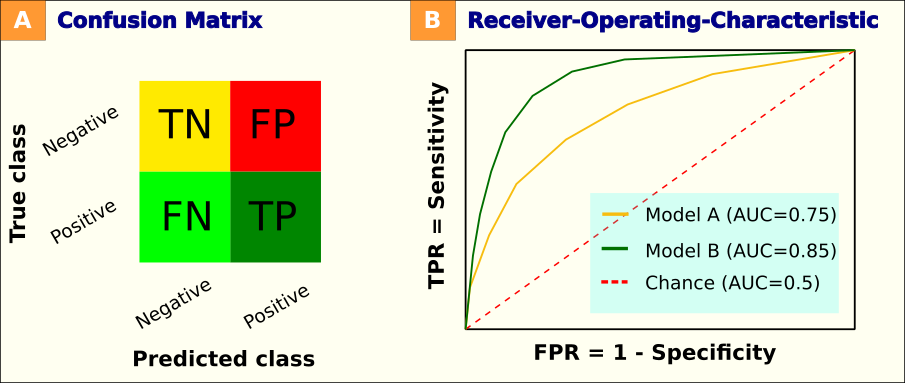


**Fig. B1.** Illustration of the performance metrics. (A) Confusion Matrix. TP: True Positive, TN: True Negative, FP: False Positive, FN: False Negative. (B) Receiver-Operating-Characteristic. TPR: True Positive Rate, FPR: False Positive Rate. AUC: Area under the Curve. Further explanations in text.

Secondary performance metrics are then calculated from these four numbers as follows:

Sensitivity = TP / (TP + FN),

Specificity = TN / (TN + FP),

Accuracy = (TP + TN) / (TP + TN + FP + FN),

Precision = TP / (TP + FP).

Generally, there is always a trade-off between Sensitivity and Specificity in modeling. This trade-off is best visualized through the concept of the ROC, displayed schematically on Fig. B1, panel B. The ROC is plotting the True Positive Rate (TPR) on the y-axis over the False Positive Rate (FPR) on the x-axis. TPR is equal to Sensitivity and FPR is equal to 1 – Specificity. The first important measure in ROC is the curve, giving an overall performance metric of the classifier at different threshold values [27]. The second important measure is given by the Area under the Curve (AUC). The AUC metric is an indicator of separability across all threshold values [27]. The threshold is a cut-off probability, according to which a model classifies samples from a test set to either one or the other group. An excellent model has an AUC near 1. A purely random classifier, e.g. throwing a coin to decide for a subject being either in the one or the other group, corresponds to AUC = 0.5 (red dashed line in Fig. 1 B). In that sense, Model B is better than Model A in the schematic illustration of the ROC (Fig. B1, panel B).

***B2. K-Fold Cross-Validation***


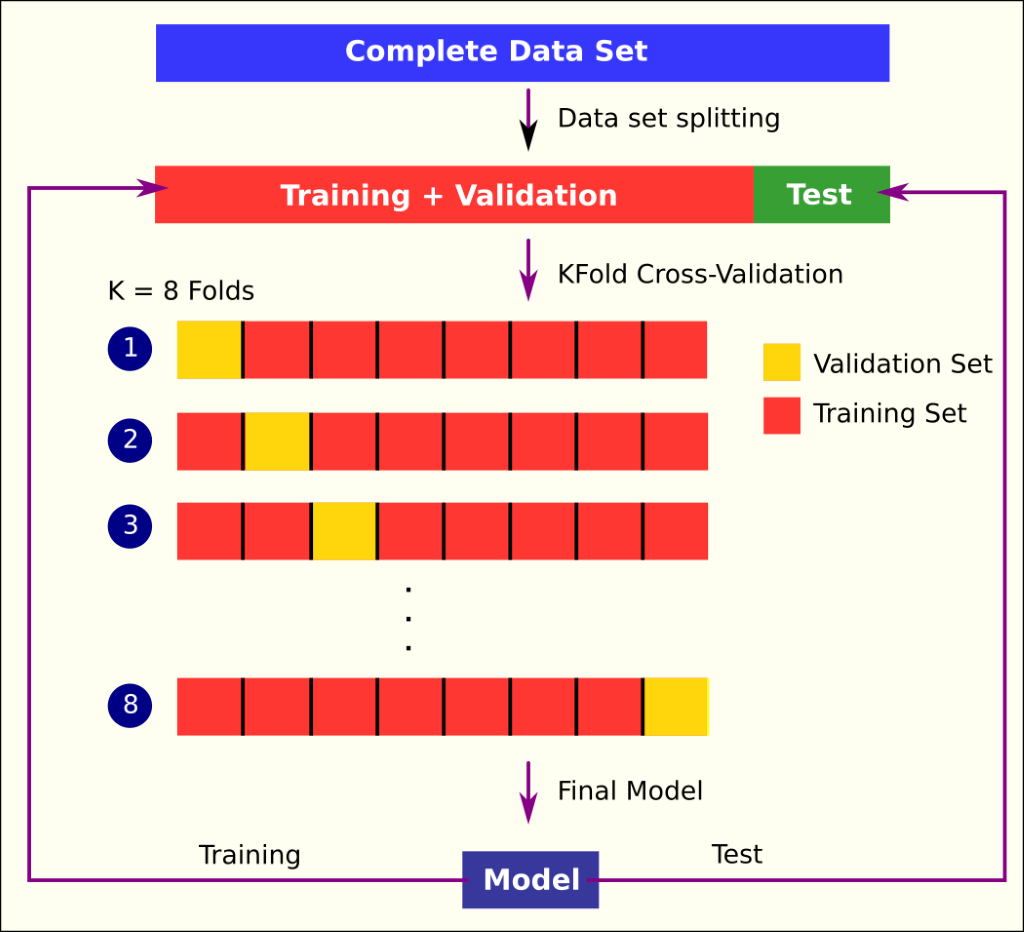


**Fig. B2.** Illustration of the full modelling cycle. Description in text.

Our modeling pipeline is illustrated in Fig. B2. After the initial splitting of the data set into a training-validation and a test subset, we performed a stratified k-fold cross-validation on the training-validation subset while the test set remained untouched to ensure later evaluation of final models. Within the k-fold cross-validation procedure, the training-validation subset was divided into K = 8 equally sized subsets. Then, a model was trained in a loop on K-1 subsets and validated on the remaining subset. On each stage of the loop, performance metrics were calculated and at the end of the loop metrics were averaged. Additionally, we calculated the average cut-off probability and corresponding cut-off fall score.

References

1. Swets JA. Indices of discrimination or diagnostic accuracy: their ROCs and implied models. Psychol Bull 1986;99(1):100-117. PMID:3704032

2. Hajian-Tilaki K. Receiver Operating Characteristic (ROC) Curve Analysis for Medical Diagnostic Test Evaluation. Caspian J Intern Med 2013;4(2):627-635. PMID:24009950

3. Metz CE. Receiver operating characteristic analysis: a tool for the quantitative evaluation of observer performance and imaging systems. J Am Coll Radiol 2006;3(6):413-422. PMID:17412096
